# The complete mitogenome of jewel click beetle *Campsosternus auratus* (Drury, 1773)

**DOI:** 10.1080/23802359.2022.2036257

**Published:** 2022-02-07

**Authors:** Xiaoning Zhang, Qingbai Hou

**Affiliations:** a College of Life Sciences, Qinghai Normal University, Xining, PR China; b Academy of Plateau Science and Sustainability, Qinghai Normal University, Xining, PR China

**Keywords:** Mitogenome, Elateridae, click beetle, *Campsosternus auratus*

## Abstract

*Campsosternus auratus* (Drury, 1773) is a large, beautiful click beetle found in Southern China. The complete mitogenome of *C. auratus* was reported in this study. The mitogenome contains 15,943 base pairs. The composition of mitogenome is 39.6% for A, 9.9% for G, 32.8% for T, and 17.7% for C. A set of 37 genes, including 13 protein-coding genes (PCGs), 22 tRNA genes, 2 rRNA genes, and one control region, were annotated. The phylogenetic result supports the monophyly of Elateridae.

Click beetles (Elateridae), the ninth most diverse family of beetles, include more than 12,000 described species worldwide (Johnson 2002), of which most are pests that can damage crops and trees having the potential to adversely impact the production of agriculture and forestry. All *Campsosternus* species are large in size and exhibit a metallic luster on their body surface, which makes them appear similar to jewel beetles (Chrysochroa). There are about 17 species and subspecies of genus *Campsosternus* reported in China. *C. auratus* is a dominant species widespread in Southern China. It is a typical species of genus *Campsosternus* (Jiang 1991), usually can be found on firs (*Cunninghamia lanceolata* (Lamb.)), grassland, or under road lamp (Jiang 2002).

Three adults *C. auratus* specimens were collected in May 2021 from Guangzhou, China (N23°9′40″, E113°22′2″). The specimens were deposited into absolute ethanol in the Insect Collection at Qinghai Normal University, Xining, China (please contact Qingbai Hou, email: bleding@126.com) under the voucher number QNU2021C000063. Total genomic DNA was extracted from the thorax muscles of a single individual using the TIANGEN Genomic DNA Extraction Kit (TIANGEN, Beijing, China) according to the manufacturer’s instructions. Four pairs of overlapped PCR primers were designed to amplify the complete mitogenome of *C. auratus*. The KOD-Plus (http://www.bio-toyobo.cn/product_detail_13.html) was used for amplification following the procedures: the initial denaturation step was performed at 95 °C for 3 mins, followed by 36 cycles reaction of 15 s at 96 °C, annealing step at 50 °C for 30 s, elongation for 3 mins at 68 °C, and the final elongation step for 5 mins at 68 °C. Then, the PCR products of amplification were collected and mixed for Next Generation Sequencing. The mitogenome was sequenced by Illumina HiSeq-PE150 with 150 bps paired-end reads at Sangon Biotech Co., Ltd., Shanghai, China. The reads were assembled with SPAdes version 3.14.1 (Bankevich et al. 2012) and GetOrganelle (Jin et al. 2020). Then the assembled mitogenome was annotated by using the MITOs WebServer (http://mitos2.bioinf.uni-leipzig.de/index.py), and manual confirmation method with reference to other Elateridae species.

The complete mitogenome of *C. auratus* is a circular DNA molecule with a length of 15,943 base pairs, and contains 37 genes, including 13 protein-coding genes (PCGs), 22 tRNA genes, 2 rRNA genes, and one control region. Its sequence was deposited in GenBank under the accession number MZ727583. The phylogenetic relationship was reconstructed based on the 13 PCGs from 25 species using the IQ-tree on XSEDE with the maximum likelihood method (Figure 1). The result highly supported the monophyly of Elateridae and showed that *C. auratus* was the sister group of *Anostirus castaneus* and *Limonius minutus*, which is consistent with the result based on 28S rDNA data (Reiko et al. 2007).

**Figure 1.**
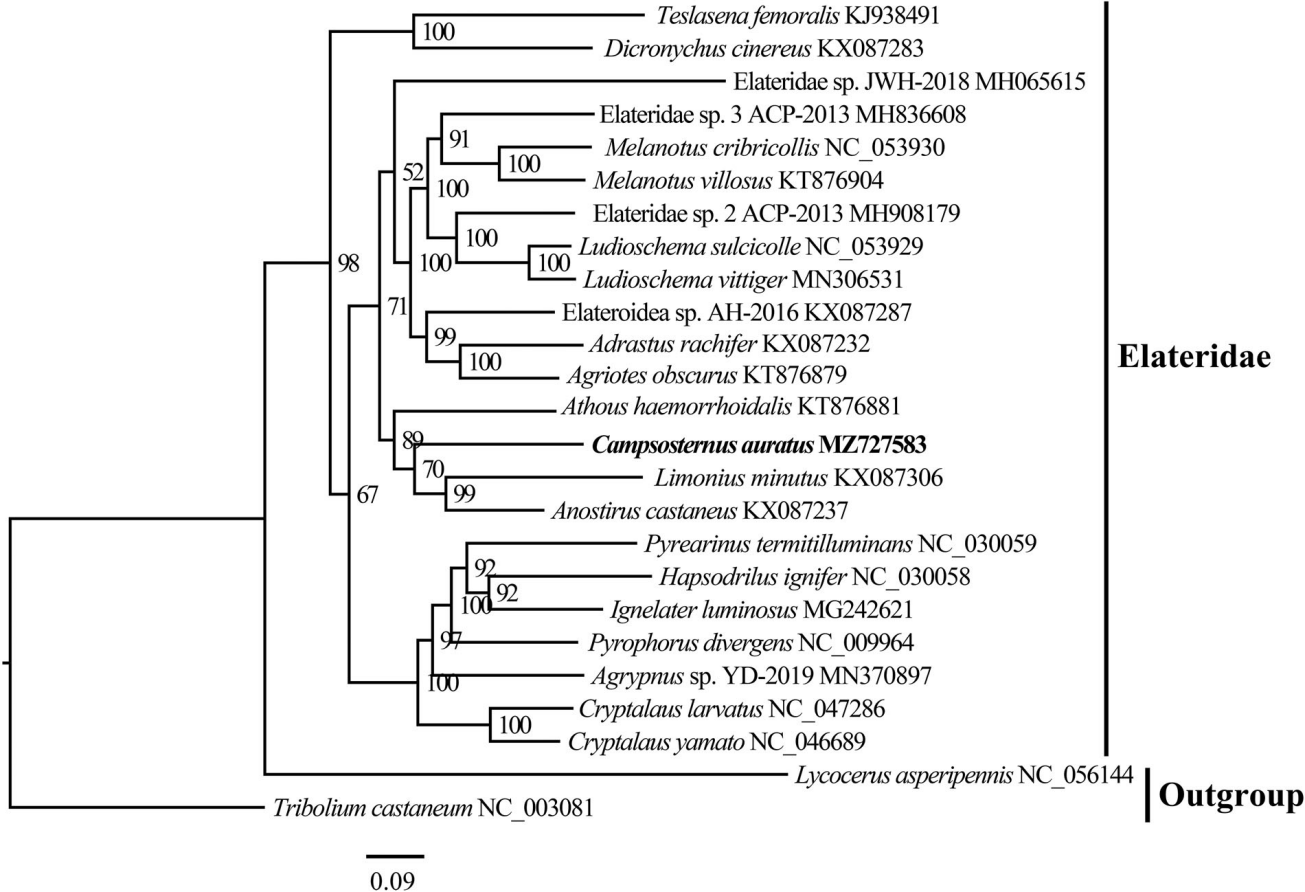
IQ-tree with the maximum likelihood method was constructed using 13 PCG sequence of *C. auratus* with 24 species of Coleoptera. The nodal numbers indicate the posterior possibility. Genbank accession numbers for the sequences are indicated next to the species names. The newly sequenced species are indicated in bold.

## Data Availability

The genome sequence data that support the findings of this study are openly available in GenBank of NCBI at (https://www.ncbi.nlm.nih.gov/) under the accession no. MZ727583. The associated BioProject, SRA, and Bio-Sample numbers are PRJNA754357, SRR15447068, and SAMN20769340 respectively.
